# Added Value of Transluminal Attenuation Gradient to Qualitative CCTA Ischemia Detection as Determined by ^13^N-ammonia PET Quantitative Myocardial Perfusion

**DOI:** 10.3390/diagnostics10090628

**Published:** 2020-08-24

**Authors:** Andrea Monroy-Gonzalez, Erick Alexanderson-Rosas, Oscar Perez-Orpinel, Magdalena Dobrolinska, Rene Tio, Jan Cees de Groot, Riemer Slart, Niek Prakken

**Affiliations:** 1 Department of Nuclear Medicine and Molecular Imaging, University Medical Center Groningen, University of Groningen, 9713 GZ Groningen, The Netherlands; magdalena.dobrolinska@gmail.com (M.D.); r.h.j.a.slart@umcg.nl (R.S.); 2 Department of Nuclear Cardiology, National Institute of Cardiology Ignacio Chavez, Mexico City 14080, Mexico; alexandersonerick@gmail.com (E.A.-R.); oscperorp@hotmail.com (O.P.-O.); 3 Department of Physiology, National Autonomous University of Mexico, Mexico City 04360, Mexico; 4 Department of Cardiology, Catharina Hospital Eindhoven, 5623 EJ Eindhoven, The Netherlands; rene.tio@catharinaziekenhuis.nl; 5 Department of Radiology, University Medical Center Groningen, University of Groningen, 9713 GZ Groningen, The Netherlands; j.c.de.groot@umcg.nl (J.C.d.G.); n.h.j.prakken@umcg.nl (N.P.); 6 Biomedical Photonic Imaging, Faculty of Science and Technology, University of Twente, 7522 NB Enschede, The Netherlands

**Keywords:** transluminal attenuation gradient, positron emission tomography, coronary computed tomography angiography, myocardial blood flow, coronary artery disease

## Abstract

Transluminal attenuation gradient (TAG) represents the slope of intraluminal contrast that decreases along a coronary vessel during coronary computed tomography angiography (CCTA). The aim of this study was to determine the added value of TAG to qualitative CCTA assessment of significant stenosis (>50%) detecting ischemia as determined by stress myocardial blood flow (MBF) or myocardial flow reserve (MFR) measured by positron emission tomography (PET). Individual contributions of TAG, qualitative assessment and the impact of calcium score were also investigated. Methods: We studied 38 consecutive patients that were referred due to suspected or known coronary artery disease (CAD). All patients underwent a two-phase hybrid ^13^N-ammonia PET/CT and CCTA. Results: TAG and presence of qualitatively assessed significant stenosis, but not calcium score, were associated with stress myocardial blood flow (MBF) and myocardial flow reserve (MFR). The area under the curves (AUC) of the linear predictor model including qualitative assessment and TAG was superior to the AUC of separate qualitative assessment or TAG for the detection of ischemia according to stress MBF (AUCs were: 88% vs. 79% and 77%; *p* = 0.01 and *p* = 0.01, respectively). Conclusions: TAG combined with qualitative CCTA assessment improved ischemia detection.

## 1. Introduction

Transluminal attenuation gradient (TAG) is a measurement representing the downslope gradient of intraluminal contrast along a coronary vessel. This diminishment of iodinated contrast concentration can be calculated by measuring Hounsfield units (HU) in the coronary arteries during coronary computed tomography angiography (CCTA) [1]. More negative values of TAG representing a steep decrease of contrast have been frequently reported in patients with coronary artery disease (CAD); therefore, TAG has been proposed as a parameter to identify hemodynamically significant stenosis [2,3,4]. 

The absence of functional information on CCTA, especially in the presence of moderate stenosis, is a limitation of CCTA [5,6]. TAG has been proposed as a simple procedure to aid the detection of significant stenosis and to increase diagnostic accuracy of CCTA, considering that its calculation can easily be performed with standard CCTA data without an additional protocol [2]. One study has reported a tendency of TAG to increase the diagnostic value of CCTA in ischemia detection as defined by ^15^O-water positron emission tomography (PET) [7]. However, it is still unknown whether TAG actually improves ischemia detection when combined with qualitative CCTA assessment and whether calcium score influences TAG parameters. Also, there is no literature comparing TAG to quantitative myocardial perfusion parameters obtained with ^13^N-ammonia PET, such as stress myocardial blood flow (MBF) and/or myocardial flow reserve (MFR). 

We aimed to determine the contributions of TAG, qualitative CCTA assessment of significant stenosis (>50%) and their combination to ischemia detection as determined by stress MBF or MFR measured by ^13^N-ammonia PET. We also aimed to investigate the influence of calcium score on these parameters.

## 2. Materials and Methods 

We retrospectively studied a consecutive list of patients who were referred to the PET/CT Unit at the National Autonomous University of Mexico, Mexico City, Mexico between January 2015 and February 2017 due to suspected or known coronary artery disease (CAD). Patients with non-ischemic cardiomyopathy and/or significant aortic valve disease were excluded. All patients signed informed consent and underwent a two-phase hybrid ^13^N-ammonia PET/CT and CCTA. The study was conducted in accordance with the standards of the local ethics committee (project number 201700840, discussed on 14 December 2017 by the Medical Ethics Review Board). For this retrospective study design, a study formal consent was waived. 

### 2.1. PET/CT Protocol

Patients were studied in a PET/CT scanner (Biograph True Point PET/CT 64-Multislice Scanner; Siemens Medical, Erlangen Germany). Myocardial perfusion was assessed at rest and during adenosine stress. Rest imaging acquisitions started with a 740 MBq ^13^N-ammonia injection. After 30 min, pharmacologic stress was performed with an injection of adenosine during a 6 min period (140 g/kg/min). A second dose of 740 MBq of ^13^N-ammonia was injected at the third minute and imaging acquisitions started immediately with the injection, as described previously [8]. Two computed tomography-based transmission scans (120 kVp; 20–30 mA; pitch 1.5) were obtained prior to the rest perfusion studies and after the stress perfusion studies for positioning and correction of photon attenuation by soft tissue, respectively. Static, dynamic and gated datasets were obtained at rest and stress for further software analysis with automated QPET software (Cedars Sinai, Los Angeles, CA, USA). Ischemia was considered as a stress MBF < 1.85 mL/g/min in any vascular territory (left anterior descending artery (LAD), left circumflex artery (LCX) and right coronary artery (RCA)) [9,10,11]. In a different analysis, ischemia was considered as MFR <2.00 in each vascular territory [9,10,11].

### 2.2. CCTA Protocol

CCTA was performed after completion of the PET acquisition. Beta-blockers were administered if heart rate was >65 beats per minute. Sublingual administration of short-acting nitrates was performed 3–4 min prior to the scan. CCTA scan was acquired with 0.60 mm collimation; gantry rotation of 330 ms; pitch of 0.2; X-ray tube current of 550–945 mAs; and tube voltage of 120 kVp. A CCTA contrast-enhanced scan was obtained after the administration of 60 to 80 mL of Iopamiron 370 IV (rate 5 mL/s) during a single breath-hold (10 s). To obtain motion-free images, standard multiphase retrospective, electrocardiography-gated and half-scan reconstruction windows were centered on the best cardiac phase image for analysis.

Two radiologists specialized in cardiovascular CCTA analyzed the images on a dedicated Leonardo workstation (Siemens, Erlangen, Germany). CCTA raw datasets were reconstructed automatically by Syngo MMWP version VE23B software. Contrast-enhanced scans were assessed using axial slices and thin-slab maximum intensity projections and were supplemented by 3D volume renderings and curved and multiplanar reformatted images if needed. Significant stenosis was defined as ≥50% coronary lumen narrowing on CCTA. Coronary calcium score was automatically determined using the Agatston score in each vessel (LAD, LCX and RCA).

### 2.3. TAG and Calcium Score

All patient CCTA images were processed in Aquarius Intuition Viewer version 4.4. This software automatically detects walls and luminal borders, vessel diameters, areas and longitudinal distances. Hounsfield units (HU) were measured within a region of interest of 1 mm^2^ at 5 mm intervals from the ostium until the cross-sectional area fell below 2.0 mm^2^. Areas with calcification were avoided (Supplementary Figure S1). TAG was calculated as the linear regression coefficient between HU and length from the ostium and considered as the change in HU per 10 mm length of each coronary artery (LAD, LCX and RCA) [3,12]. Segments with blooming artefacts caused by coronary calcium were excluded from the linear regression [13] (Supplementary Figure S1). Vessels with an inadequate image quality due to excessive motion artefacts that would not allow following the lumen of a coronary artery, coronary stent or total occlusion were excluded.

### 2.4. Statistical Analysis

Categorical variables are shown as simple proportions. Data are presented as the mean ± standard deviation or as the median and interquartile range (IQR). Spearman correlation coefficient was used to determine correlations between continuous variables. Student *t*-test or Mann–Whitney U test were used to compare continuous data. The generalized estimating equation (GEE) was used to account for repeated measurements in a linear model. The best correlation matrix was chosen according to the quasi-likelihood under the independence model criterion and the Wald test was used without correction for multiple comparisons. A *p*-value < 0.05 was considered statistically significant. Predicted values of GEE models combining qualitative assessment by CCTA and TAG were obtained to predict quantitative myocardial perfusion parameters. Receiver operating characteristics (ROC) curves and area under the curves (AUC) were calculated in order to obtain optimal cut-off values. AUCs were compared by the DeLong method [14] with Bonferroni correction (*p*-value < 0.0166 was considered statistically significant). Sensitivity, specificity, positive predictive value (PPV) and negative predictive value (NPV) were also calculated. Statistical analyses were performed using IBM SPSS Statistics for Windows, version 23.0 (IBM Corp., Armonk, NY, USA) and Medcalc for Windows, version 19.2.1 (MedCalc Software, Ostend, Belgium).

## 3. Results

Of 43 patients who underwent a two-phase hybrid ^13^N-ammonia PET/CT, CCTA and coronary calcium quantification, four patients were excluded due to non-ischemic cardiomyopathy and one due to a stent in each of the three main coronary arteries. Baseline characteristics of the remaining 38 patients are summarized in Table 1. One hundred one vessel territories (34 LAD, 34 LCX and 33 RCA) remained for analysis after exclusion of 13 vessels due to inadequate image quality. 

### 3.1. PET/CT

Among 101 vessels, mean rest MBF was 0.8 ± 0.3 mL/g/min, mean stress MBF was 2.3 ± 0.7 mL/g/min, and mean MFR was 3.0 ± 1.1. Twenty-eight percent of the vascular regions had ischemia according to stress MBF and 20% had ischemia according to the MFR.

### 3.2. CCTA and Calcium Score

From the 101 coronary vessels, 23% presented a significant stenosis on CCTA. Segments with ischemia according to stress MBF showed a higher proportion of significant stenosis on the CCTA than those without ischemia (64% (*n* = 18) vs. 7% (*n* = 5), *p* < 0.001). Similar results were observed when ischemia was considered according to MFR (74% (*n* = 14) vs. 11% (*n* = 9), *p* < 0.001). Median calcium score was 1 (0–114). Patients with ischemia according to stress MBF showed higher median calcium score than those without ischemia (103 (0–448) vs. 0 (0–28), *p* = 0.002). Similar results were seen when ischemia was considered according to MFR (122 (46–533) vs. 0 (0–26), *p* < 0.001). Perfusion parameters, TAG and calcium score along different stenosis levels are shown in Table 2. 

### 3.3. Transluminal Attenuation Gradient

Mean TAG of all included vessels was −11 ± 9. There was a statistically significant difference of TAG among patients with and without ischemia according to stress MBF or MFR (−16 ± 8 vs. −9 ± 8, *p* < 0.001 and −17 ± 10 vs. −10 ± 8, *p* < 0.001, respectively). Also, there was a statistically significant difference of TAG among patients with and without significant stenosis found on the CCTA (−16 ± 9 vs. −9 ± 8, *p* = 0.001). TAG correlated negatively with coronary calcium score (*r* = −0.35, *p* < 0.001) and positively with stress MBF and MFR (*r* = 0.36, *p* < 0.001 and *r* = 0.40, *p* < 0.001, respectively). 

GEE models demonstrated that qualitative assessment and TAG, but not calcium score, were significantly associated with stress MBF and MFR (Table 3 and Table 4). We did not find any confounding effect or interaction between TAG and calcium score for the prediction of stress MBF or MFR. A sensitivity analysis was performed using only patients without a previously diagnosed MI showing similar results (Supplementary Tables S1 and S2). We obtained similar results in a model using only qualitative assessment and TAG to predict stress MBF and MFR of all 101 vessels (Supplementary Tables S3 and S4), and the model-predicted values of the linear predictors were saved in this step of the analysis.

**Figure 1:**
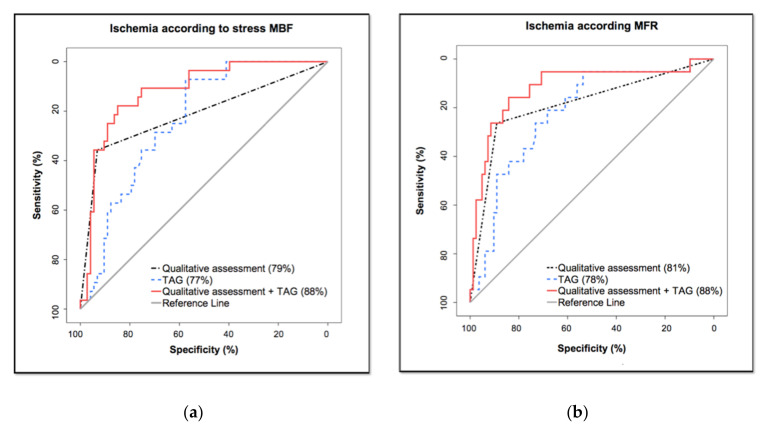
Receiver operating characteristic curve analysis comparing TAG and qualitative assessment of significant stenosis (≥50%) to ischemia according to: (**a**) stress MBF; (**b**) MFR.

ROC curve demonstrated that the diagnostic accuracy of qualitative assessment for the detection of significant stenosis by CCTA and TAG for the detection of ischemia as determined by stress MBF and MFR measured by PET/CT was similar (AUCs were: 79% vs. 77%, *p* = 0.81, and 81% vs. 78%, *p* = 0.65, respectively). However, the AUC of the model including both qualitative assessment and TAG was superior to the AUCs of qualitative assessment alone and TAG alone for the detection of ischemia according to stress MBF (AUC was 88% vs. 79% and 77%; *p* = 0.01 and *p* < 0.01, respectively). The diagnostic accuracy of the model including both qualitative assessment and TAG was similar to the AUC of qualitative assessment alone and superior to the AUC of TAG alone for the detection of ischemia according to MFR (AUC was 88% vs. 81% and 78%; *p* = 0.10 and *p* = 0.0161, respectively) (Figure 1). Optimal cut-off point for the diagnosis of ischemia in a vascular territory according to both stress MBF and MFR was a TAG of ≤−10. Sensitivity, specificity, PPV and NPV, of TAG, qualitative assessment of stenosis by CCTA, and the combination of both methods are shown in Supplementary Tables S5 and S6).

## 4. Discussion

This study compares the diagnostic value of TAG, qualitative CCTA stenosis assessment and their combined performance for accurate ischemia detection as determined by ^13^N-ammonia PET. The model combining TAG and qualitative assessment compared to stress MBF was superior to single TAG or qualitative assessment. However, the model combining TAG and qualitative assessment predicting MFR was only superior to TAG. The use of solely TAG or qualitative assessment showed similar diagnostic value for ischemia detection as determined by stress MBF and/or MFR. While TAG was lower in patients with ischemia according to stress MBF or MFR and significant stenosis was also more common these patients, it is interesting that only one of each four patients with ischemia according to stress MBF and one of each three patients with ischemia according to MFR presented with a stenosis according to qualitative CCTA assessment. These results suggest that both TAG and visual assessment of stenosis provide complementary information that only in combination improves ischemia detection. Because stress MBF quantification marks hemodynamically significant coronary stenosis while perfusion reserve is more related to the prognosis of patients with different degrees of CAD [15,16,17], our results may indicate that TAG is associated with ischemia due to flow limiting stenosis and possibly cardiovascular prognosis. One interesting finding is that while the presence of coronary calcium was higher in patients with ischemia, TAG remained a predictor of ischemia after adjustment for coronary calcium. 

The results of this study are partially in agreement with Bom et al. [7]. Similar to our study, TAG alone did not significantly increase the AUC over CCTA alone for the diagnosis of ischemia according to stress MBF ^15^O-water PET or fractional flow reserve [7]. In addition, our results show that the combination of both measurements significantly (*p* = 0.01) improved the detection of ischemia, in comparison to Bom et al. who reported only a trend (*p* = 0.053). Because the study of Bom et al. was performed with a 256-slice CT scanner (Brilliance iCT, Philips Healthcare, Best, The Netherlands) [7], the current study also demonstrates that their results can similarly be extrapolated to a 64-Multislice scanner and ischemia according to stress MBF measured by ^13^N-ammonia PET. Benz et al. has previously described the relationship between corrected coronary opacification and ^13^N-ammonia PET [18]. Their results suggest that attenuation of contrast in the coronary artery may be of value in the diagnostic workup of coronary stenosis. Meanwhile, other studies using fractional flow reserve (FFR) also show similar results regarding the diagnostic value of CCTA and TAG for ischemia [3,13,19,20]. From these studies, Stuijfzand et al. [13] and Xu et al. [20] have reported that excluding the presence of calcified segments did not alter the diagnostic accuracy of TAG. Similarly, our study can conclude that coronary calcium score may not affect the association between TAG and stress MBF. To our best knowledge, we are the first study to report an improvement of the diagnostic value when incorporating TAG and qualitative CCTA assessment into a single model for ischemia detection as determined by ^13^N-ammonia PET. 

While controversy remains around TAG being an adequate functional computed tomography parameter, it is interesting that it has a similar diagnostic accuracy as qualitative CCTA assessment. Currently there are many questions regarding TAG such as whether it is a cost-effective method for the detection of coronary artery stenosis, or whether TAG could have positive implications for patients with otherwise non-evaluable segments, for example, in patients with severe calcification, coronary motion artifacts, stair-step artifacts and in patients with intermediate stenosis. Our results suggest that even after excluding non-evaluable segments, such as those with calcification, TAG remains a predictor of quantitative myocardial perfusion. This may be due to TAG being a measurement derived of different points along the vessel, and not depending on one specific qualitatively diseased area, which may make it a more reliable tool. 

When automated, we think TAG could possibly gain clinical value, especially if it could be implemented in computer aided diagnosis to complement qualitative CCTA assessment, increasing total diagnostic performance. It will be important to clarify whether TAG can properly work as a functional parameter of the coronary vasculature and whether it may also have prognostic value. Therefore, prospective studies are needed to completely understand the diagnostic value of TAG and whether reclassification based on TAG may increase the diagnostic accuracy of PET/CCTA hybrid imaging. Furthermore, a better understanding of the relationship of coronary calcium score and TAG as well as the diagnostic value of TAG with single beat scans is needed.

This study has several limitations. It is a retrospective study with a relatively small sample size. Our population was studied in a 64-Multislice scanner, which may have affected the quality of images for the assessment of TAG. Branches (posterior descending and diagonals) were not assessed, which could have influenced diagnostic performance of the TAG. We also cannot rule out that blooming artefacts did not affect measurements despite our efforts to avoid calcified areas while measuring TAG [13,20]. TAG was not corrected by contrast opacification (CCO) of the corresponding descending aortic opacification; while it has been reported that TAG and TAG-CCO appear to be interchangeable measurements [13], we cannot exclude that this has influenced our results. Furthermore, we cannot exclude the possibility that TAG is influenced by the vessel diameter or length [2,20,21]. Finally, we could not compare our results to invasive assessment and we do not have a record of the number of patients that received a PET guided intervention, such as percutaneous coronary intervention or coronary artery bypass.

## 5. Conclusions

The combination of TAG with qualitative assessment of significant stenosis using CCTA shows improved ischemia detection in this proof of concept study using stress MBF ^13^N-ammonia PET as the gold standard. Especially with the development of a tool for (semi) automatic calculation, TAG could represent a complementary tool in accurately assessing coronary artery stenosis. 

## Figures and Tables

**Table 1:** Baseline characteristics of patients.

Characteristics	All Patients (*n* = 38)
Age (years)	65 ± 13 years
Male gender	28 (74%)
Diabetes mellitus	13 (34%)
Hypertension	24 (63%)
Dyslipidemia	20 (53%)
Smoker	15 (39%)
Body mass index	27 ± 4
Left ventricle ejection fraction in rest	63 ± 14
Left ventricle ejection fraction in stress	65 ± 11
Previous MI	12 (32%)
Right coronary artery dominance	36 (95%)

**Table 2:** Perfusion parameters, transluminal attenuation gradient (TAG) and calcium score along different stenosis levels.

	Stress MBF	MFR	TAG	Calcium Score
0–25% stenosis (*n* = 58)	2.4 (2.1–2.8)	3.3 (2.4–3.9)	−8 (−12–−3)	0 (0–0)
26–50% stenosis (*n* = 20)	2.4 (1.9–2.7)	3.3 (3.0–3.6)	−10 (−14–−9)	52 (5–169)
51–70% stenosis (*n* = 19)	1.6 (1.4–1.8)	1.4 (1.1–3.2)	−13 (−18–−10)	249 (106–533)
71–99% stenosis (*n* = 4)	1.2 (0.9–1.8)	1.9 (1.0–2.8)	−25 (−45–−13)	506 (10–1239)

**Table 3:** Association between stress myocardial blood flow (MBF) and variables. Multivariate analysis.

	Beta	Lower 95% CI	Upper 95% CI	*p*-Value
Constant	2.61	2.45	2.77	<0.001
Stenosis ≥ 50% on qualitative assessment	−0.79	−1.16	−0.43	<0.001
Transluminal attenuation gradient	0.02	0.01	0.03	<0.01
Calcium score	0.00	0.00	0.00	0.38

**Table 4:** Association between myocardial flow reserve (MFR) and variables. Multivariate analysis.

	Beta	Lower 95% CI	Upper 95% CI	*p*-Value
Constant	3.58	3.18	3.98	<0.001
Stenosis ≥ 50% on qualitative assessment	−0.96	−1.65	−0.26	<0.01
Transluminal attenuation gradient	0.03	0.00	−0.05	0.02
Calcium score	0.00	0.00	0.00	0.47

## Supplementary Materials

The following are available online at https://www.mdpi.com/2075-4418/10/9/628/s1. Figure S1: (A) Coronary computed tomography angiography of a left anterior descending coronary artery (LAD) with calcifications along the vessel. (B) Same LAD shows cross-sectional areas in which calcifications were found; areas above the green arrows had eccentric calcifications; therefore, Hounsfield units (HU) were measured avoiding such areas; areas above red crosses were excluded due to blooming artefacts caused by calcification. (C) Correlation between HU and length; segments with calcification are shown as dots in green and red, corresponding to areas that were included and excluded from the analysis, respectively. (D) Cross-sectional area of a segment with eccentric calcification that was included in the analysis. (E) Cross-sectional area of a segment with blooming artefact excluded from the analysis. (F) Correlation between HU and length after excluding red dots in Figure 1C. Table S1: Generalized estimating equation model showing the best predictors of stress MBF after excluding patients with myocardial infarction. Table S2: Generalized estimating equation model showing the best predictors of MFR after excluding patients with myocardial infarction. Table S3: Generalized estimating equation model showing the best predictors of stress MBF. Table S4: Generalized estimating equation model showing the best predictors of MFR. Table S5: Sensitivity, specificity, PPV and NPV, of TAG, visual detection of stenosis by CCTA and combination of both methods when compared to ischemia defined by stress MBF. Table S6: Sensitivity, specificity, PPV and NPV, of TAG, visual detection of stenosis by CCTA and combination of both methods when compared to ischemia defined by MFR.
