# Supplementary material for: Added Value of Transluminal Attenuation Gradient to Qualitative CCTA Ischemia Detection as Determined by 13N-ammonia PET Quantitative Myocardial Perfusion
Source: Diagnostics (Basel). 2020 Aug 24;10(9):628. doi: 10.3390/diagnostics10090628 (PMC7555852; doi:10.3390/diagnostics10090628)
Supplement: Supplementary file 1 [file diagnostics-10-00628-s001.pdf]

diagnostics

Supplementary material

Added Value of Transluminal Attenuation Gradient to Qualitative CCTA Ischemia Detection as Determined by ^{13}N-ammonia PET Quantitative Myocardial Perfusion

Andrea Monroy-Gonzalez [1,*], Erick Alexanderson-Rosas [3,4], Oscar Perez-Orpinel [3], Magdalena Dobrolinska [1], Rene Tio [5], Jan Cees de Groot [2], Riemer Slart [1,6] and Niek Prakken [2]

[1] Department of Nuclear Medicine and Molecular Imaging, University Medical Center Groningen, University of Groningen, 9713 GZ, Groningen, The Netherlands; a.monroy.gonzalez@umcg.nl (A.M.-G); magdalena.dobrolinska@gmail.com (M.D.); r.h.j.a.slart@umcg.nl (R.S.)
[2] Department of Radiology, University Medical Center Groningen, University of Groningen, 9713 GZ, Groningen, The Netherlands; j.c.de.groot@umcg.nl (J.C.d.G.); n.h.j.prakken@umcg.nl (N.P.)
[3] Department of Nuclear Cardiology, National Institute of Cardiology Ignacio Chavez, Mexico City, 14080, Mexico; alexandersonerick@gmail.com (E.A.-R.); oscperorp@hotmail.com (O.P.-O)
[4] Department of Physiology, National Autonomous University of Mexico, Mexico City, 04360, Mexico.
[5] Department of Cardiology, Catharina Hospital Eindhoven, 5623 EJ, Eindhoven, The Netherlands; rene.tio@catharinaziekenhuis.nl
[6] Biomedical Photonic Imaging, Faculty of Science and Technology, University of Twente, 7522 NB, Enschede, The Netherlands.
* Correspondence: a.monroy.gonzalez@umcg.nl

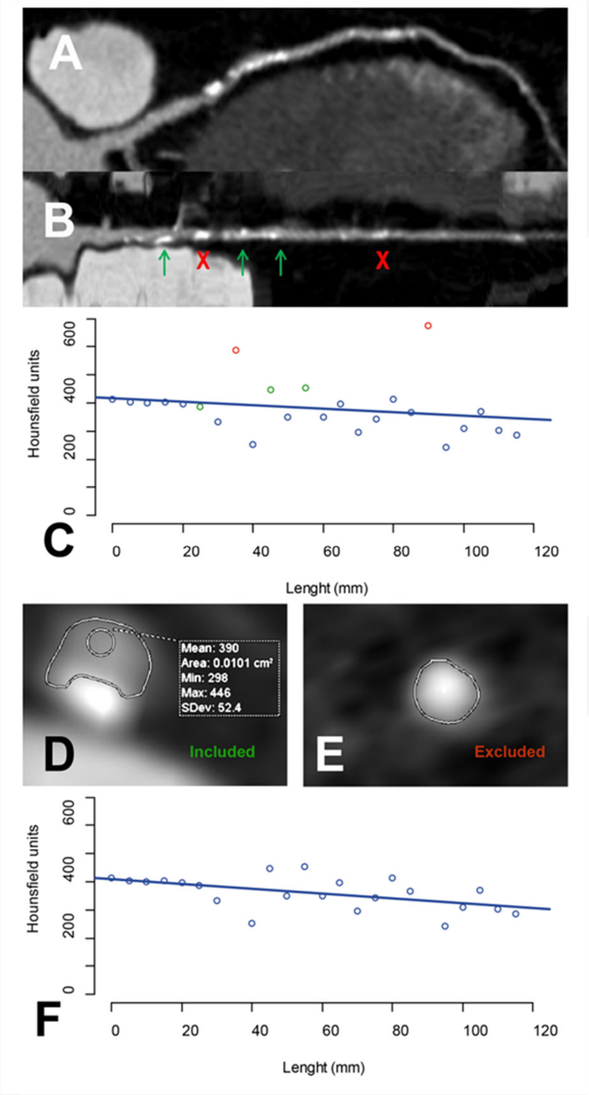

Supplementary Figure S1. (**A**) Coronary computed tomography angiography of a left anterior descending coronary artery (LAD) with calcifications along the vessel. (**B**) Same LAD shows cross-sectional areas in which calcifications were found; areas above the green arrows had eccentric calcifications, therefore, Hounsfield Units (HU) were measured avoiding such areas; areas above red crosses were excluded due to blooming artefacts caused by calcification. (**C**) Correlation between HU and length; segments with calcification are shown as dots in green and red, corresponding to areas that were included and excluded from the analysis, respectively. (**D**) Cross-sectional area of a segment with eccentric calcification that was included in the analysis. (**E**) Cross-sectional area of a segment with blooming artefact excluded from the analysis. F) Correlation between HU and length after excluding red dots in Figure 1C.

Supplementary Table S1. Generalized estimating equation model showing the best predictors of stress MBF after excluding patients with myocardial infarction.

	Beta	Lower 95% CI	Upper 95% CI	p value
Constant	2.64	2.48	2.79	<0.001
Stenosis ≥ 50% on qualitative assessment	−0.60	−1.09	−0.10	0.02
Transluminal attenuation gradient	0.02	0.00	0.03	0.03
Calcium score	0.00	0.00	0.00	0.81

Supplementary Table S2. Generalized estimating equation model showing the best predictors of MFR after excluding patients with myocardial infarction.

	Beta	Lower 95% CI	Upper 95% CI	p value
Constant	3.56	3.14	3.98	<0.001
Stenosis ≥ 50% on qualitative assessment	−0.92	−1.91	−0.07	0.07
Transluminal attenuation gradient	0.03	0.00	0.06	0.04
Calcium score	0.00	0.00	0.00	0.71

Supplementary Table S3. Generalized estimating equation model showing the best predictors of stress MBF.

	Beta	Lower 95% CI	Upper 95% CI	p value
Constant	2.60	2.44	2.77	<0.001
Stenosis ≥ 50% on qualitative assessment	−0.71	−1.00	−0.41	<0.01
Transluminal attenuation gradient	0.02	0.01	0.03	<0.01

Supplementary Table S4. Generalized estimating equation model showing the best predictors of MFR.

	Beta	Lower 95% CI	Upper 95% CI	p value
Constant	3.59	3.18	3.99	<0.001
Stenosis ≥ 50% on qualitative assessment	−1.07	−1.64	0.51	<0.001
Transluminal attenuation gradient	0.03	0.01	0.05	0.01

Supplementary Table S5. Sensitivity, specificity, PPV and NPV, of TAG, visual detection of stenosis by CCTA and combination of both methods when compared to ischemia defined by stress MBF.

Stress MBF	Sensitivity	Specificity	PPV	NPV	Accuracy
TAG	93%	58%	46%	95%	67%
CCTA	64%	93%	78%	87%	85%
Predicted value TAG + CCTA	86%	75%	57%	93%	78%

Supplementary Table S6. Sensitivity, specificity, PPV and NPV, of TAG, visual detection of stenosis by CCTA and combination of both methods when compared to ischemia defined by MFR.

MFR	Sensitivity	Specificity	PPV	NPV	Accuracy
TAG	95%	52%	32%	98%	60%
CCTA	74%	89%	61%	94%	86%
Predicted value TAG + CCTA	95%	71%	43%	98%	75%

© 2020 by the authors. Submitted for possible open access publication under the terms and conditions of the Creative Commons Attribution (CC BY) license (http://creativecommons.org/licenses/by/4.0/).
